# Co-expression IL-15 receptor alpha with IL-15 reduces toxicity via limiting IL-15 systemic exposure during CAR-T immunotherapy

**DOI:** 10.1186/s12967-022-03626-x

**Published:** 2022-09-27

**Authors:** Ying Zhang, Qinghui Zhuang, Fang Wang, Can Zhang, Chang Xu, Aiqin Gu, William H. Zhong, Yi Hu, Xiaosong Zhong

**Affiliations:** 1 The Clinical Center of Gene and Cell Engineering, Beijing Shijitan Hospital, Capital Medical University, Haidian District, No. 10, Iron Medicine Road, Yang Fang Dian, Beijing, 100038 China; 2 Carriage Therapeutics for Affiliation, Beijing, China; 3 River Hill High School, Clarksville, MD, USA

**Keywords:** Armored chimeric antigen receptor T, Interleukin 15, Interleukin 15 receptor alpha, Toxicity

## Abstract

**Background:**

Chimeric antigen receptor (CAR)-T cell therapy is a powerful adoptive immunotherapy against both B-cell malignancies and some types of solid tumors. Interleukin (IL) -15 is an important immune stimulator that may provide ideal long-term persistent CAR-T cells. However, higher base line or peak serum IL-15 levels are also related to severe toxicity, such as cytokine release syndrome (CRS), graft-versus-host disease (GVHD), and neurotoxicity.

**Methods:**

We successfully constructed CD19 specific armored CAR-T cells overexpressing IL-I5 and IL-15 receptor alpha (IL-15Ra). In vitro cell differentiation and viability were monitored by flow cytometry, and an in vivo xenograft mouse models was used to evaluate the anti-tumor efficiency and liver damage of CAR-T cells.

**Results:**

CAR-T cells overexpressing IL-15 alone demonstrated enhanced viability, retarded exhaustion in vitro and superior tumor-inhibitory effects in vivo. However, these tumor-free mice had lower survival rates, with serious liver injuries, as a possible result of toxicity. As expected, CAR-T cells overexpressing IL-15 combined with IL-15Ra had reduced CD132 expression and released fewer cytokines (IFNγ, IL-2 and IL-15) in vitro, as well as had the tendency to improve mouse survival via repressing the growth of tumor cells and keeping livers healthier compared to CAR-IL-15 T cells.

**Conclusions:**

These results indicated the importance of IL-15 in enhancing T cells persistence and IL-15Ra in reducing the adverse effects of IL-15, with superior tumor retardation during CAR-T therapy. This study paves the way for the rapid exploitation of IL-15 in adoptive cell therapy in the future.

**Supplementary Information:**

The online version contains supplementary material available at 10.1186/s12967-022-03626-x.

## Background

Owing to its great ability to expand and stimulate T cells, IL-2 functions as the first cytokine to be used in clinical cancer trials. However, the administration of IL-2 leads to T cells exhaustion limiting its application, especially in adoptive T-cell therapy. Recently, IL-15 has drew considerable attention in cytokine receptor biology because of its similarity to IL-2. Studies have reported that the administration of recombinant IL-15 or over-expression of IL-15 protects mice from various infections [1, 2], and due to its ability to prolong T cell longevity, the clinical use of IL-15 has been further strengthened.

As members of the common γ-chain family cytokines, both IL-15 and IL-2 are involved in the regulation of T cell homeostasis and differentiation. These cytokines share many biological activities, presumably because of similarity with the IL-2 receptor complex [3]. The shared activities include growth and migration of activated T and natural killer (NK) cells, as well as induction of B cell differentiation and proliferation [4]. IL-15 and IL-2 each utilize a heterotrimeric receptor complex, consisting of IL-2Rβ and γc subunits and a specific, unique α subunits [3]. IL-15 receptor alpha (IL-15Ra) is an IL-15-specific receptor with a high affinity. Following secretion by immune cells, IL-15 binds to IL-15Ra expressed on effector cells and this IL-15-IL-15Ra fusion protein is then bound to the IL-2Rβγ (IL-15Rβγ) complex expressed on nearby effector cells, such as the NK, B, and T cells, in turn, leading to the recruitment and activation of Janus kinase 1 (JAK1) and JAK3 [5]. Activated JAK1 and JAK3 further prompt the transcription of IL-15-regulated genes in the effector cells [6] via signal transducer and activator of transcription 3 (STAT3) and STAT5. In addition, IL-15 can also bind to the IL-15Rβγ complex in the absence of IL-15Ra, resulting in the activation of the PI3K and MAPK pathways [7]. Thus, IL-15 and IL-15-IL-15Ra complexes contribute differently to T-cell-mediated immune responses.

Chimeric antigen receptor (CAR) is a synthetic molecules that is constructed by an extracellular tumor antigen-binding domain, hinge, transmembrane, and intracellular signaling domains [8]. T cells expressing CAR (CAR-T cells) can directly recognize tumor-associated antigens through a single-chain variable fragment (scfv) of the extracellular domain. After recognition, CAR-T cells are activated to release multiple cytokines, such as granzyme, perforin, and interferon-γ (IFN-γ), thereafter inducing apoptosis of tumor cell [9, 10]. CAR-T cell development involves four stages. First-generation CAR, which dose not contain a co-stimulatory signal domain, was developed to treat cancers but with poorly persistence in vivo. To solve this problem, second-generation CAR was incorporated with co-stimulatory signal domains from CD28 or 4-1BB into the CAR intracellular structure. Since the two domains determine different functional properties of CAR-T cells, a third generation of CARs containing CD28 and 4-1BB was established [11]. However, despite the significant breakthrough in CAR-T therapy, in terms of its clinical curative effects, several factors still limit the anti-tumor efficacy of CAR-T cells. For example, the exhaustion of CAR-T cells is universal [12], which leads to poor anti-tumor activity and relapse. Thus, the fourth generation CAR, which is also called armored CAR, based on the third-generation CAR combined with CRISPR/Cas9 technology, cytokine, antibody, etc. [13, 14] was constructed to overcome these deficiencies of CAR-T cells.

There is ample pre-clinical and clinical evidence demonstrating that CAR-T cells are predisposed to exhaustion and poor persistence, which limits the efficacy of immunotherapy [11]. The application of IL-15 has been reported to solve this problem to some extent. However, other adverse events, such as toxicity, can occur. CAR-T cells can cause toxicity via several mechanisms. If the tumor-associated antigen to which the CAR is targeted is expressed on normal tissues, those tissues may be damaged, as is the case in which normal B cells are damaged and depleted by CD19 CAR-T cells [15]. In addition, the most prominent and well-described toxicity of CAR-T cells is cytokine release syndrome (CRS), which is caused by cytokines released by infused T cells with symptoms including hypotension and fever. Neurological toxicities may also occur due to CAR T-cell therapy [16, 17]. Notably, the application of IL-15, which is reported to induce a better anti-tumor response, is always accompanied by serious toxicity [18, 19].

In this study, to reduce the adverse effects and amplify the advantages of IL-15 during CAR-T therapy, we generated CD19 specific armored CARs connecting IL-15 and IL-15Ra. We found that the combination of IL-15Ra and IL-15 could block the toxicity but had no effect on the persistence and anti-tumor activity of CAR-T cells induced by IL-15. This findings provides clues for the rapid application of IL-15 in the treatment of cancer patients during adoptive cell therapy.

## Materials and methods

### Cell lines

The human NALM-6, and the retrovirus packaging cell line PG13 were purchased from the American Type Culture Collection (ATCC). The NALM-6 cell expressing eGFP and firefly luciferase was generated by retroviral infection. NALM-6 cell was maintained in RPMI-1640 (Lonza) and containing 10% fetal bovine serum (Biosera) and 10,000 IU/mL penicillin/10,000 μg/mL streptomycin (EallBio Life Sciences). All cells were cultured in 5% CO_2_, 95% air at 37 °C in a humidifed incubator.

### Generation of CD19 specific CAR-T cells

CD19 Specific CAR coding sequence was synthesized by GeneArt (Invitrogen) and then subcloned into the SFG retroviral vector. The cDNAs of IL-15 (HG10360-M) and IL-15Ra (HG18366-G) were purchased from Sino Biological. All cloning of the CARs were verified by sequencing. PG13 cells were used to produce retroviral particles after plasmid transient transfection. Human peripheral blood mononuclear cells (PBMCs) from healthy donors were isolated by Lymphoprep (MP Biomedicals) gradient centrifugation. After stimulated with anti-CD3/CD28 beads, T cells from PBMCs were then infected with retrovirus. After 7 days, the CAR-T cells were subjected to CAR expression detection and then expanded in X-VIVOTM15 serum-free medium containing 5% GemCellTM Human Serum AB with IL-2 (138 U/ml). This research was approved by the Beijing Shijitan Hospital Institutional Review Board and informed consent was obtained from all the healthy donors.

### Flow cytometry

Flow cytometry was performed on a FACSCanto Plus instrument (BD Biosciences) and FlowJo v.10 (FlowJo, LLC) was used for data analysis. All antibodies were purchased from BD Biosciences. CAR-T cells were detected after staining with APC-cy7-labeled mouse anti-human CD3 antibody, FITC-labeled mouse anti-human CD8 antibody, Alexa Fluor 700-labeled mouse anti-human CD8 antibody, BV421 labeled mouse anti-human CD4 antibody, BV605-labeled mouse anti-human CD45RO, PE-cy7-labeled mouse anti-human CCR7, Alexa Fluor 700-labeled mouse anti-human CD27, PE-cy5-labeled mouse anti-human CD95, and Alexa Fluor 647-labeled goat anti-mouse IgG (Fab specific) F(ab′)2 antibody (Jackson ImmunoResearch).

### Western blot analyses

Cells were washed with PBS three times and then the protein was extracted using RIPA buffer. The protein samples were quantified using a Pierce BCA Protein Assay Kit (Thermo Fisher Scientific), and then denatured in sodium dodecyl sulfate (SDS)/β-mercaptoethanol sample buffer. Samples were separated on a 15% SDS–polyacrylamide gel and blotted onto polyvinylidene fluoride membranes (Millipore) by electrophoretic transfer. The membrane was incubated with rabbit anti-human IL-15Ra antibody (Sino Biological) overnight at 4 °C, and then the specific protein-antibody complex was detected using HRP-conjugated goat anti-rabbit secondary antibody (Santa Cruz Biotechnology). Detection of the chemiluminescence reaction was carried out using an ECL kit (Thermo Fisher Scientific).

### Cytotoxicity assay

CAR-T cells were co-cultured with or without NALM-6-eGFP (2:1) in a 24-well plate. After 24 h, cells were collected and tumor cells were detected by surface markers using flow cytometry (BD FacsCanto II Plus) per the manufacturer’s instructions.

CAR-T cells were co-cultured with or without NALM-6-eGFP (2:1) in a 96-well plate. After 24 h, the luciferases activities were monitored using the IVIS imaging system (IVIS, Xenogen, Alameda, CA, USA) after adding 20 μL D-luciferin potassium (1.515 mg/mL) (Thermo Fisher Scientific). Cell viability = fluorescence value of live cells/control × 100%

### Proliferation assay

CD19-CAR-T cells, CD19-CAR-IL-15 T cells and CD19-CAR-IL-15-IL-15Ra T cells were cultured with NALM-6-eGFP (10:1). The cell number at the day 0, day 7 and day 14 were counted by Viable through Trypan blue exclusion using the Vi-CELL Cell Viability Analyzer.

### Analysis of cytokine production

The CAR-T cells were co-cultured with NALM-6-eGFP at an E:T ratio of 2:1 for 24 h. The supernatants were collected and subjected to IFN-γ, IL-15 and IL-2 detection via ELISA kits (DY285B, D1500, DY202, R&D systems) according to the manufacturer’s instructions.

### PCR

IL-15 and IL-15Ra expression in antigen-stimulated CAR-T cells was confirmed by PCR. Total RNAs were extracted from CAR-T cells using TRIzol Reagent (Invitrogen) following the manufacturer’s instructions. cDNA was synthesized using the High Capacity cDNA Reverse Transcription kit (Thermo Fisher Scientific). The expression of IL-15 gene was amplified using primers 5′- ATGGATGCAATGAAGAGAGGG-3′ (sense) and 5′- CGACGTGTTCATGAACATCTGGA-3′ (antisense); IL-15Ra was amplified using primers 5′-ATGGCCCCGAGGCGGGCGCGAGG-3′ (sense) and 5′- TAGGTGGTGCGAGCAGT-3′ (antisense); GAPDH was amplified using primers 5′- TGACCACAGTCCATGCCATC-3′ (sense) and 5′-GTGAGCTTCCCGTTCAGCTC-3′ (antisense) functions as the control.

### Xenograft mouse model with injection of NALM-6-eGFP cells

Six- to eight- week-old NOD-SCID mice were purchased from Charles River Laboratories. Then, 1 × 10^6^ NALM-6-eGFP cells were injected into NOD-SCID mice intravenously to generate a xenograft mouse model. One days after the tumor cell injection, 1 × 10^7^ CAR-T cells (2 × 10^6^ CAR positive T cells) were injected into the tail vein once a day for 3 days. Tumor development was monitored using an IVIS. When imaged, 200 μL D-luciferin potassium (15.15 mg/mL) was injected intraperitoneally. As the negative control, non-transduced T cells (NT) were injected intravenously. The mice were monitored for over three months. All experiments, including those with mice, were approved by the Institutional Review Board of Beijing Shijitan Hospital.

### Haematoxylin–eosin (H&E) staining

The mice were monitored over a period of time. When the mice died, liver samples excised from the mice were fixed in 4% paraformaldehyde. The H&E staining was performed by Bioss company (Beijing, China).

### Graft-versus-host disease (GVDH) scoring

GVHD scoring was performed according to Hechinger et al. [20] in detail, liver tissue was assessed for bile duct injury (manifested by nuclear hyperchromasia, nuclear crowding, infiltrating lymphocytes, and cytoplasmic vacuolation) and inflammation (infiltration with lymphocytes, neutrophils, and eosinophils). Disease was scored between 0 and 4 based on the number of involved tracts and the severity of disease in each tract (0, none; 1, few involved tracts with mild involvement; 2, numerous involved tracts but with only mild disease; 3, injury in the majority of tracts; and 4, severe involvement of most tracts).

### Statistical analyses

Graphs and statistical analyses were performed using GraphPad Prism, version 8.0.2. The data were analyzed using a Student *t* test followed by parametric test or Mann–Whitney test with *p* values < 0.05 considered as statistically significant. For the measurement of cytokines using ELISA, data are shown as means ± S.E.M. of at least three independent experiments, each performed in triplicate. For all flow cytometric results, data are shown as means ± S.D. of at least three independent experiments. The overall survival of mice with NALM-6-eGFP xenografts was measured using the Mental-Cox test for group comparison. *p < 0.05; **p < 0.01; ***p < 0.001; ****p < 0.0001; n.s., not significant.

## Results

### Armored CD19 specific CAR-T cells are developed

**Fig. 1:**
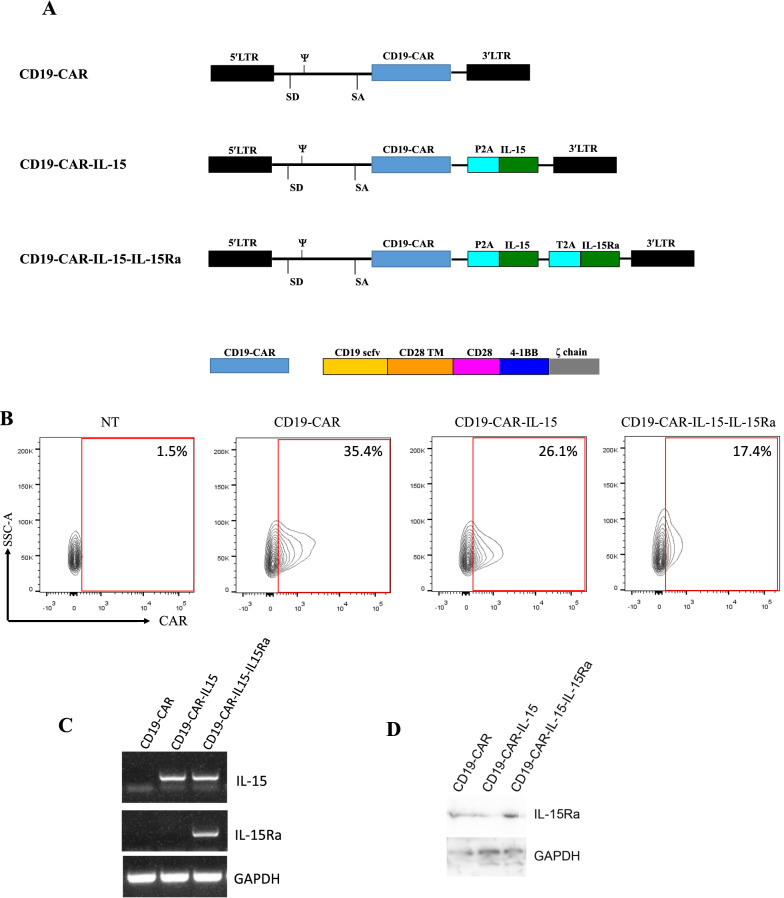
CD19 specific armored CAR-T cells are developed. **A** Schematic diagram of third different CD19 specific CARs. It consists of the third generation CD19-CAR, P2A, IL-15 and IL-15Ra. **B** Flow cytometry was used to detect the expression of CD19 specific CARs by using goat anti-mouse IgG (Fab specific). **C** Total RNA was extracted from different CD19-CAR-T cells to detect the relative expression of IL-15, IL-15Ra via PCR. **D** Total proteins were extracted from different CD19-CAR-T cells to detect the expression of IL-15Ra. SD, splice donor; SA, splice acceptor. NT, non-transduced

IL-15 and IL-15Ra genes connected to the CD19-CAR gene were constructed (Fig. 1A) and these co-expressing retroviral vector were transduced into T cells. Figure 1B shows that the transduction efficiency, and CD4/CD8 ratios were similar among the three groups (Additional file 1: Fig. S1). Next, successful expressions of IL-15 and IL-15Ra were confirmed using PCR. Total RNAs were extracted from the CAR-T cells. It was shown that CD19-CAR-IL-15 T cells overexpressed IL-15 and CD19-CAR-IL-15-IL-15Ra T cells overexpressed IL-15 as well as IL-15Ra (Fig. 1C). The expression of IL-15Ra was also confirmed by western blotting (Fig. 1D).

### IL-15 armored CAR-T cells exhibit higher proliferative and less-differentiated phenotype in vitro

**Fig. 2:**
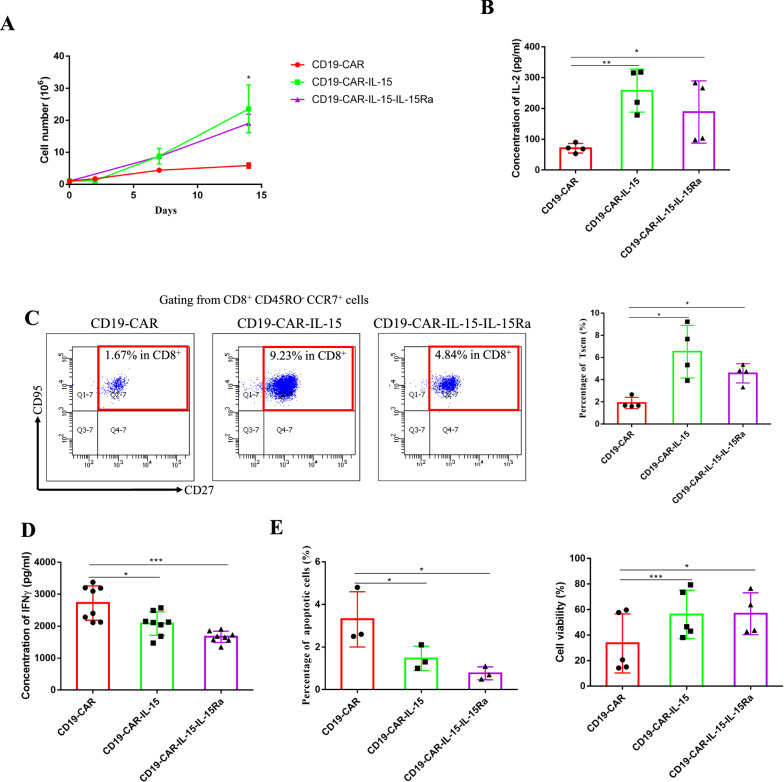
IL-15 armored CAR-T cells exhibit higher proliferative and less-differentiated phenotype in vitro. **A** The extent of T cell proliferation was reflected through direct cell counting over time. CAR-T cells were stimulated every 7 days with fresh NALM-6-eGFP cells (10:1), and T cells were counted before the addition of NALM-6-eGFP cells. **B** CAR-T cells were co-cultured with NALM-6-eGFP (2:1) without IL-2 in the culture medium for 24 h. The concentration of IL-2 in the supernatant was measured via ELISA. **C** CAR-T cells were co-cultured with NALM-6-eGFP (10:1) for 7 days, the T subsets were detected using flow cytometry. **D** CAR-T cells were co-cultured with NALM-6-eGFP (2:1) for 24 h. The supernatant was collected to detect the concentration of IFN-γ via ELISA. **E** CAR-T cells were co-cultured with NALM-6-eGFP (10:1). After 7 days, all cells were collected and the percentages of apoptotic cells and cell viability were detected through Annexin V and 7-ADD staining. Results were analyzed by student’s *t*-test followed by parametric test. * *p* < 0.05; ** *p* < 0.01; *** *p* < 0.001

The percentage of CAR positive T cells was adjusted to be consistent using non-transduced T cells. Direct cell counting showed that IL-15 and IL-15Ra overexpressed CAR-T cells exhibited higher proliferation capacity after NALM-6-eGFP stimulation (Fig. 2A). In addition, as IL-2 is a growth factor for T cells, the concentrations of IL-2 in the supernatant were measured and it was shown that CD19-CAR-IL-15 and CD19-CAR-IL-15-IL-15Ra T cells released more IL-2 than CD19-CAR T cells (Fig. 2B). The differentiated phenotypes of CAR-T cells were determined. After stimulation with NALM-6-eGFP cells for 7 days, results showed that only 1.67% of CD8^+^ CD19-CAR T cells were T-memory stem cells (Tscm), whereas there were more Tscm cells among the CD19-CAR-IL-15 and CD19-CAR-IL-15-IL-15Ra T cells (9.23% and 4.84% respectively) (Fig. 2C and Additional file 1: Fig. S2). In addition, it has been reported that less differentiated T cells produce less IFNγ upon antigen stimulation. Thus, CD19-CAR, CD19-CAR-IL-15 and CD19-CAR-IL-15-IL-15Ra T cells were stimulated with NALM-6-eGFP cells for 24 h and the concentration of IFNγ was measured using ELISA. As showed in Fig. 2D, IL-15 and IL-15Ra exhibited T cells with less IFNγ production, implying a less-differentiated phenotype for CD19-CAR-IL-15 and CD19-CAR-IL-15-IL-15Ra T cells. Subsequently, as IL-15 amplifies T cell proliferation, the percentages of apoptotic cell and cell viability were analyzed. We found that IL-15 and IL-15Ra suppressed CAR-T cells apoptosis and enhanced cell viability (Fig. 2E).

### IL-15Ra reduces the IL-15-induced toxicity in vitro

**Fig. 3:**
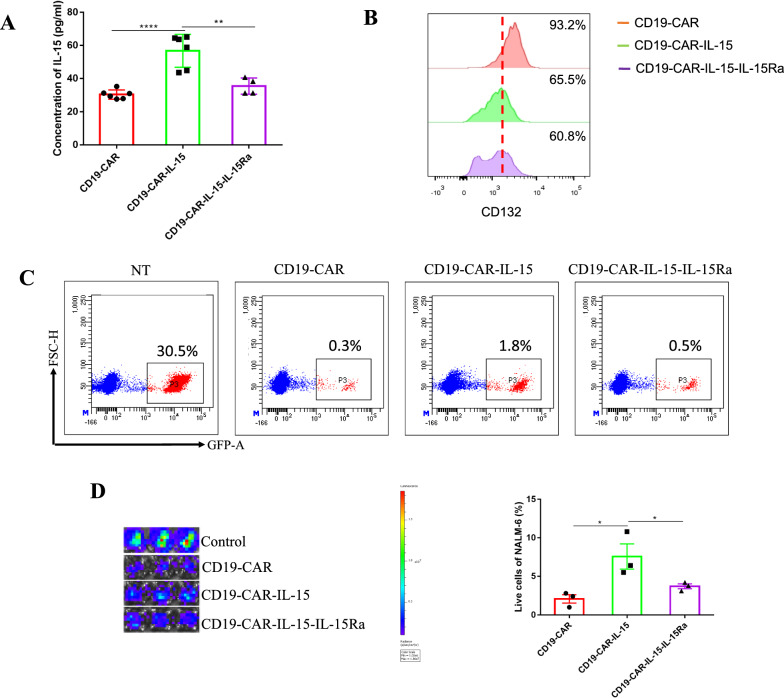
IL-15Ra reduces the IL-15-induced toxicity in vitro. **A** CAR-T cells were co-cultured with NALM-6-eGFP (2:1) for 24 h. The supernatant was collected to detect the concentration of IL-15 via ELISA. **B** CAR-T cells were co-cultured with NALM-6-eGFP (10:1) for 7 days, the expression of CD132 on the cell surface was detected using flow cytometry. **C** CAR-T cells were co-cultured with NALM-6-eGFP cells (2:1) in 24-well plate for 24 h. Flow cytometry was used to detect the live NALM-6-eGFP. **D** CAR-T cells were co-cultured with NALM-6-eGFP cells (2:1) in 96-well plate for 24 h. Luciferase activity was used to determine the tumor cell viability. Left panel shows the picture obtained from IVIS imaging system, right panel shows the statistical analysis. Control represents NALM-6-eGFP cells. Results were analyzed by student’s *t*-test followed by parametric test. * *p* < 0.05; ** *p* < 0.01; *** *p* < 0.001

To study the influence of IL-15Ra on IL-15 under cell culture condition, the supernatants of armored CAR-T cells were collected to detect the concentration of IL-15 using ELISA. CD19-CAR-IL-15 T cells showed the highest IL-15 release, whereas CD19-CAR-IL-15-IL-15Ra T cells release the same levels as CD19-CAR-T cells. As shown in Fig. 1C, CAR-IL-15-IL-15Ra T cells successfully expressed IL-15 and IL-15Ra, demonstrating that IL-15Ra combines with IL-15 to reduce the concentration of IL-15 in the medium, which has the potential to reduce toxicity. In addition, an IL-15 receptor, CD132, with a high expression related to GVHD [20], was detected. Figure 3B shows that CD19-CAR-IL-15-IL-15Ra T cells have the lowest CD132 expression (60.8% compared with 93.2% for CD19-CAR and 65.5% for CD19-CAR-IL-15, respectively). The anti-tumor activity of armored CAR-T cells was also investigated. CD19-CAR T cells and CAR-IL-15-IL-15Ra T cells had the same anti-tumor capacity, while CD19-CAR-IL-15 T cells had the lowest (Fig. 3C and D).

### IL-15 armored CAR-T cells co-expressed with IL-15Ra exhibit enhanced anti-tumor activity and reduced toxicity in vivo

**Fig. 4:**
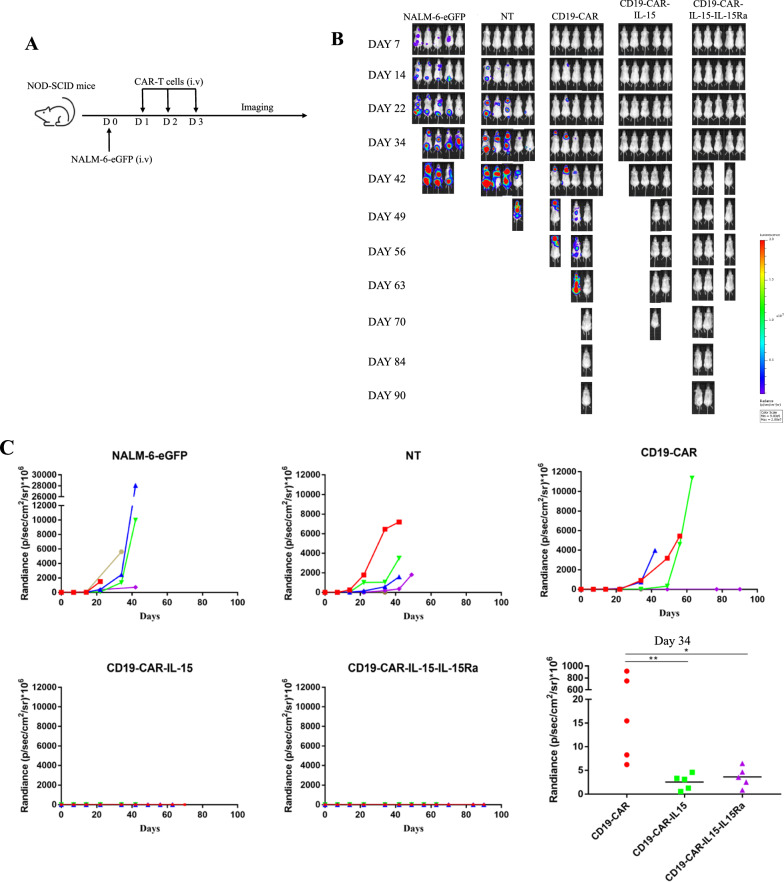

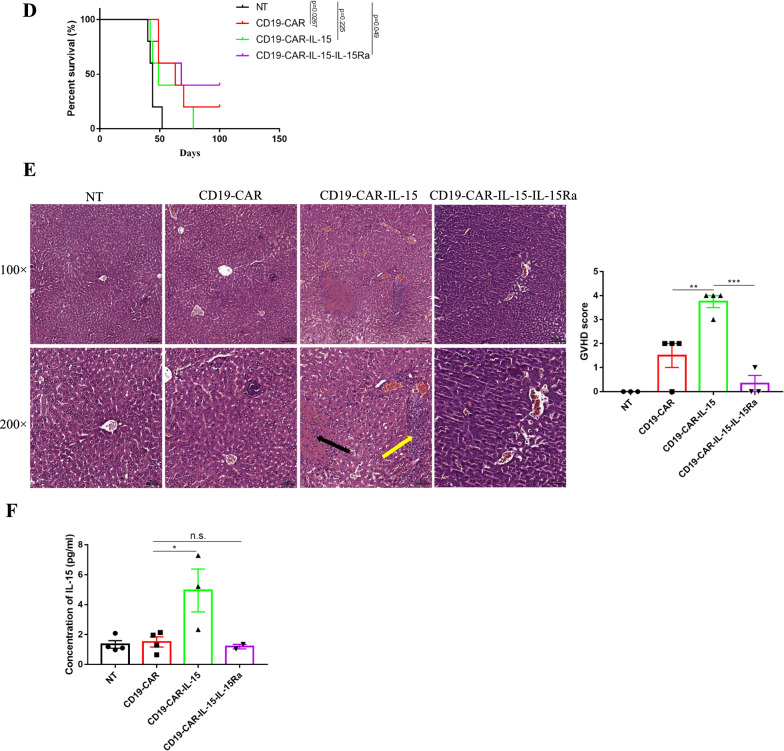
IL-15 armored CAR-T cells expressed with IL-15Ra exhibit enhanced anti-tumor activity and reduced toxicity in vivo. **A** Schematic diagram of mouse experimental processes. 1 × 10^6^ NALM-6-eGFP cells were injected into NOD-SCID mice intravenously to construct the xenograft mouse model. One days after tumor cell injection, 1 × 10^7^ CAR-T cells (2 × 10^6^ CAR positive cells) were injected into tail vein once a day for 3 days. Tumor development was monitored using IVIS. **B, C** Quantitative bioluminescence (radiance = photons/cm^2^/sr) imaging data for all mice are shown. Statistic analysis of quantitative bioluminescence of day 34 is shown. **D** Overall survival rates of mice with NALM-6-eGFP xenografts are shown. **E** Livers from CAR-T treated mice were collected to stain hematoxylin and eosin. Yellow arrow shows the numerous of infiltrated neutrophils, the black arrow shows the area of necrotic lesions. Right panel shows the GVHD scores of the livers. **F** Sera from mice were extracted and the concentrations of human IL-15 were measured using ELISA. Results were analyzed by student’s *t*-test followed by parametric test or Mann–Whitney test. The survival curve was analyzed using Mantel-Cox test. * *p* < 0.05; ** *p* < 0.01; *** *p* < 0.001; n.s., not significant

To further examine the anti-tumor activity of armored T cells in vivo, NALM-6-eGFP cells were injected intravenously into NOD-SCID mice to generate a xenograft mouse model. After one day, T cells were injected intravenously with non-transduced T cells (NT) as the negative control, and the mice were monitored for more than three months (Fig. 4A). As shown in Fig. 4B, C and Additional file 1: Fig. S3B, compared with control mice with a rapid progression of tumor development, mice treated with CD19-CAR-IL-15 T cells and CD19-CAR-IL-15-IL-15Ra T cells showed no tumor development, implying enhanced anti-tumor activity induced by IL-15. Despite no tumor development, the survival rate of the CD19-CAR-IL-15 T cell treated group was the lowest compared with the CD19-CAR and CD19-CAR-IL-15-IL-15Ra T cell-treated groups, in which all mice died within 70 days (Fig. 4B and D). Compared with the CD19-CAR group, in which only 20% of the mice lived for more than 90 days, the survival rate of the CD19-CAR-IL-15-IL-15Ra group was 40% (Fig. 4B and D). Most importantly, the livers were collected when the mice died to observe duct injury and inflammation via H&E staining. Figure 4E and Additional file 1: Fig. S3A show that the livers of CD19-CAR-IL-15 T-treated mice had severe structural abnormalities, with numerous neutrophils infiltrating in the portal area and a large area of necrotic lesions in the lobules, and those of CD19-CAR-IL-15-IL-15Ra T-treated mice were relatively healthy. The GVHD score was approximately 4 for CD19-CAR-IL-15 T-treated mice and 0 for the other three groups, demonstrating that IL-15Ra reduced the adverse effects of IL-15. Before the mice died, bloods from each mouse were collected and the sera were used to detect the concentration of human IL-15. As shown in Fig. 4F, CD19-CAR-IL-15 T-treated mice had the highest levels of human IL-15 in the blood, implying that the co-expression of IL-15Ra blocked the density of IL-15 in the serum. These results indicate that IL-15Ra had the tendency to prolonged survival rate via reducing IL-15 toxicity.

## Discussion

In the present study, we evaluated the effects of IL-15 and IL-15-IL-15Ra complex on CAR-T cell therapy. Our results demonstrated that IL-15 armored CAR-T cells resulted in the highest percentage of Tscm cells and cell viability in cell culture condition. The combination of IL-15 and IL-15Ra had a higher percentage of Tscm despite lower than IL-15 alone. IL-15-IL-15Ra armored CAR-T cells showed the lowest toxicity with less cytokine release (IFNγ) and CD132 expression. In a xenograft mouse model, IL-15 armored CAR-T cells completely inhibited tumor relapse but with severe toxicity (all mice died within 70 days with severe liver injury), whereas IL-15-IL-15Ra armored CAR-T cells completely inhibited tumor relapse with lower toxicity (40% of mice surviving for more than 90 days with health liver). Importantly, the GVHD score of the livers from CD19-CAR-IL-15 T-treated mice was approximately 4, whereas that of the livers from CD19-CAR-IL-15-IL-15Ra T-treated mice was approximately 0, demonstrating the inhibitory effect of IL-15Ra on the IL-15 toxicity.

CD19 specific CAR-T therapy has demonstrated significant anti-tumor effects in B cell malignancies and some types of solid tumors [21]. However, various studies have shown that CAR-T products generated by different methods or from different laboratories exhibit inconsistent anti-tumor efficiency. Several factors limit the anti-tumor efficiency of CAR-T cells, such as antibody affinity, off-target toxicity, impressive tumor microenvironment as well as terminal differentiation [22]. CAR-T cell persistence is a crucial problem and considerable progress has been made in obtaining cell products that result in enhanced expansion, persistence, and anti-tumor response. Studies have shown anti-tumor benefits for using IL-15 as well as IL-7 in culture [23] or IL-15 genetically engineered into T cells with CAR synchronously [24, 25]. Furthermore, high IL-15 levels in the serum of patients with DLBCL and B-ALL were associated with better outcomes after CD19 CAR-T therapy [26, 27]. These outcomes demonstrate that the combined adjuvant IL-15 improved the anti-tumor efficacy of CAR-T therapy. Indeed, in this study we developed an armored CAR for the treatment of leukemia. Our in vitro cell cultivation results indicated that this armored CAR-targeting CD19 increased cell viability, inhibited apoptosis and maintained CAR-T cells with a less differentiated phenotype, demonstrating the enhanced longevity of T cells [28, 29].

Although several clinical studies have reported that elevated IL-15 expression correlates with improved patient survival, the administration of IL-15 has caused considerable toxicity including hypotension, fever, and thrombocytopenia in cancer patients, which may further prevent the safety approval of IL-15 [18, 30]. In line with these studies, using the NALM-6 tumor treated mouse, we found IL-15 armored CD19-CAR-T cells completely inhibited tumor relapse; however, the toxicity was serious that all mice died within 70 days with serious liver damage and higher GVHD score, indicating a severe adverse effect of IL-15. The IL-15-IL-15Ra complex has been shown to significantly stimulate CD8^+^ T cells, especially memory CD8^+^ T cells, to enhance cytotoxicity against multiple tumors, such as myeloma [31], breast cancer [32], colorectal carcinoma [33], and prolonged survival of tumor-bearing mice, establishing a long-term immune memory against tumor re-challenge [34, 35]. A fusion protein of IL-15-IL-15Ra and anti-FAP caused superior targeted anti-tumor killing ability, providing a rationale for the development of antibody-IL-15-IL-15Ra fusion proteins for cancer immunotherapy in the future [36]. Thus, in the present study, after using IL-15Ra as a part of the CAR cassette, our study found that overexpression of IL-15Ra together with IL-15 enhanced cell viability, reduced apoptotic cells and retained T cell differentiation with more Tscm compared with conventional CAR-T cells. Finally, IL-15Ra inhibited tumor development completely in tumor-treated mice with relatively health livers and lower GVHD score compared with CD19-CAR-IL-15 T cells, providing a new option for improving CAR construct.

IL-15 cis presentation by IL-15Ra expressed on CD8^+^ T cells was also able to enhance the proliferation and viability of these CD8^+^ T lymphocytes in vivo [37] and IL-15Ra significantly increased the stability of IL-15 in serum [38]. In one study, peripheral T cells stably expressing second-generation CD19-CAR and IL-15-IL-15Ra retarded leukemia development and sustained resistance after tumor clearance with long-lived Tscm [24]. However, this did not indicate a toxicity effect of IL-15. In our study, we found that IL-15 combined with IL-15Ra reduced adverse events significantly during CAR-T therapy that had the potential ability to prolong the survival rate of tumor-treated mice with the health liver and lower GVHD score, indicating a novel function of IL-15Ra. There are two reasons for this finding. Firstly, the CD19 specific CAR we used was the third generation of CARs with CD28 and 4-1BB into the CAR intracellular structure, which may influence intracellular signal transduction [39]. Second, unlike previous studies, we constructed CD19-specific armored CARs that connected CAR, IL-15 and IL-15Ra into the same plasmid. Therefore, the co-expression of CD19-CAR and IL-15-IL-15Ra fusion proteins within a single cells may influence the function of IL-15Ra. Nevertheless, the mechanism underlying the reduced toxicity induced by IL-15Ra requires further research.

CD132, a common γ chain, is a subunit of interleukin receptors including IL-2, IL-4, IL-7, IL-9 and IL-15. Because the levels of these cytokines were shown to be increased in the serum of patients developing acute and chronic GVHD and inhibition of CD132 could have a profound effect on GVHD [20], CD132 expression on the armored CAR-T cells was examined, and the expression level of CD132 was the lowest for CAR-T cells together with IL-15 and IL-15Ra compared with CD19-CAR-T and CD19-CAR-IL-15 T. In addition, studies have shown that inflammatory bowel disease is associated with increased soluble CD132 expression [40], and increased expression of IL-7Rα together with that of CD132 is positively related to psoriasis-like skin inflammation [41]. Therefore, for the IL-15-IL-15Ra complex armored CAR-T cells, low-toxicity towards patients after application could be possible predicted.

Toxicities caused by CAR-T cells are varied and not fully understood; thus, the administration of immunosuppressive agents to decrease toxicity is an evolving practice [42, 43]. IL-15 stimulates the proliferation and activation of various immune cells, especially CD8^+^ T cells, leading to increased cytotoxicity and production of cytokines, but also amplifies the adverse effects of CAR-T cells [44], such as severe GVHD. Herein, using CD19-specific CAR, our results indicate that IL-15 combined with IL-15Ra reduced the expression of CD132 compared to conventional CAR-T and IL-15 armored CAR-T cells, leading to the relatively highest survival rate and keeping liver healthier in tumor-treated mice. Even so, the novel application of IL-15Ra in clinic may be not consistent with in xenograft mouse model [45]. This research provides a preliminary study of IL-15Ra and the clinical application needs a long way to go. In addition, it has been reported that the application of IL-15 during CAR-T therapy is beneficial for various targets, such as VEGFR-2 [29], Her-2 [46], glypican-3 [47], etc. In this project, we used CAR targeted CD19 cells only to study the effect of IL-15Ra, which is a limitation of this study. In future, this model will be applied to other CARs for further research.

## Conclusions

In summary, sustaining the Tscm population during ex vivo expansion prior to adoptive T-cell therapy is challenging. Here, we demonstrate that the combination of IL-15 for CAR-T cell generation preserves the Tscm phenotype and results in enhanced self-renewal capacity but with severe toxicity in vivo. Next, we successfully constructed a CD19 specific CAR combined with IL-15-IL-15Ra fusion proteins and examined its cell viability in vitro and immunotoxicity in xenograft mouse models, which provide a candidate tool for clinical leukemia treatment.

## Supplementary Information

**Additional file 1: Figure S1.** CAR-T cells were subjected to flow cytometry to detect the expression of CD4 and CD8. **Figure S2.** CAR-T cells were subjected to flow cytometry to detect the Tscm. **Figure S3.** 1 × 10^6^ NALM-6-eGFP cells were injected into NOD-SCID mice intravenously to construct the xenograft mouse model.

## Data Availability

The original data of the study are available from the corresponding authors upon reasonable request.
