# Supplementary material for: Co-expression IL-15 receptor alpha with IL-15 reduces toxicity via limiting IL-15 systemic exposure during CAR-T immunotherapy
Source: J Transl Med. 2022 Sep 27;20:432. doi: 10.1186/s12967-022-03626-x (PMC9516829; doi:10.1186/s12967-022-03626-x)
Supplement: Supplementary file 1 — Additional file 1: Figure S1. CAR-T cells were subjected to flow cytometry to detect the expression of CD4 and CD8. Figure S2. CAR-T cells were subjected to flow cytometry to detect the Tscm. Figure S3. 1 × 10^6^ NALM-6-eGFP cells were injected into NOD-SCID mice intravenously to construct the xenograft mouse model. [file 12967_2022_3626_MOESM1_ESM.docx]

**Additional file Fig. 1.** CAR-T cells were subjected to flow cytometry to detect the expression of CD4 and CD8**.**

**
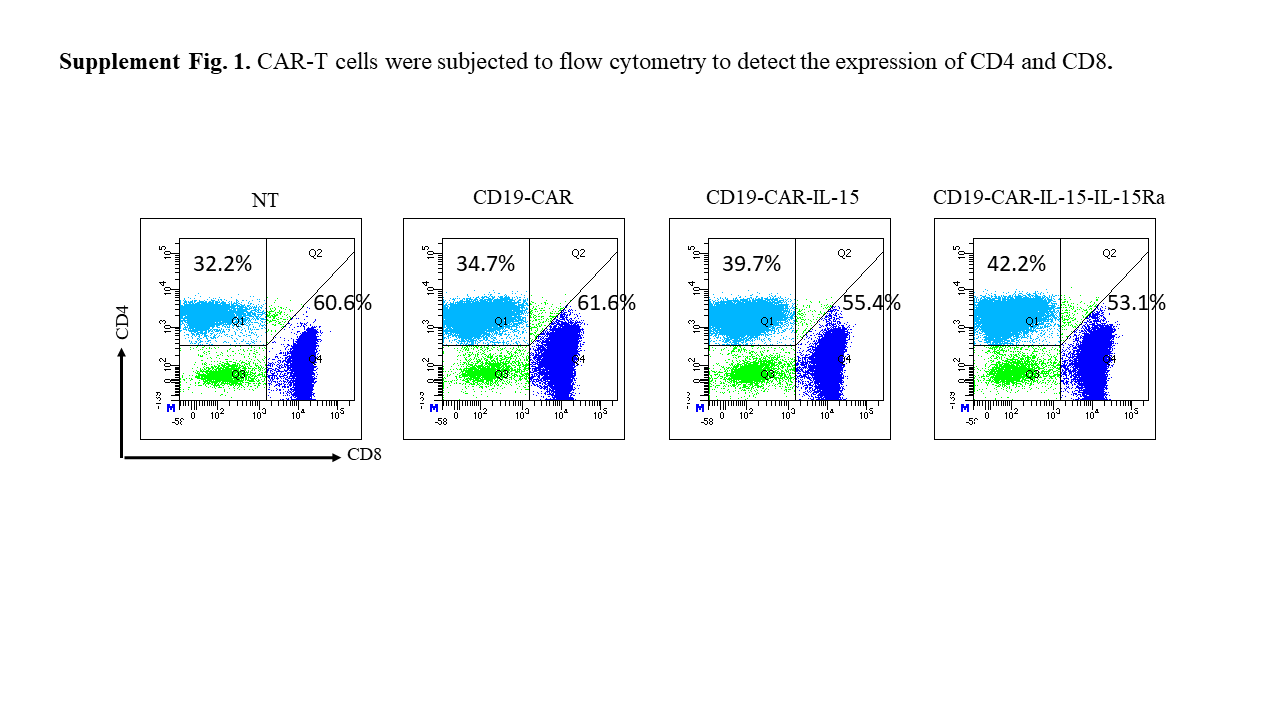
**

53.1%

**Additional file Fig. 2.** CAR-T cells were subjected to flow cytometry to detect the Tscm.


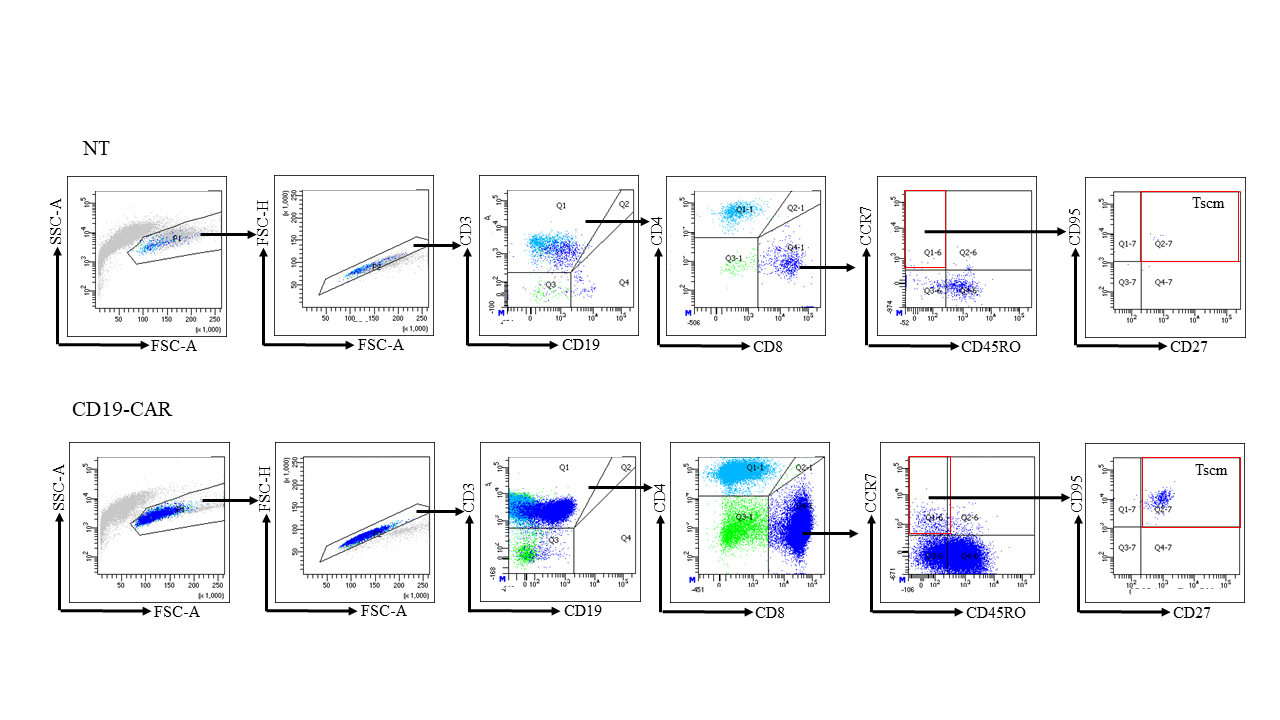


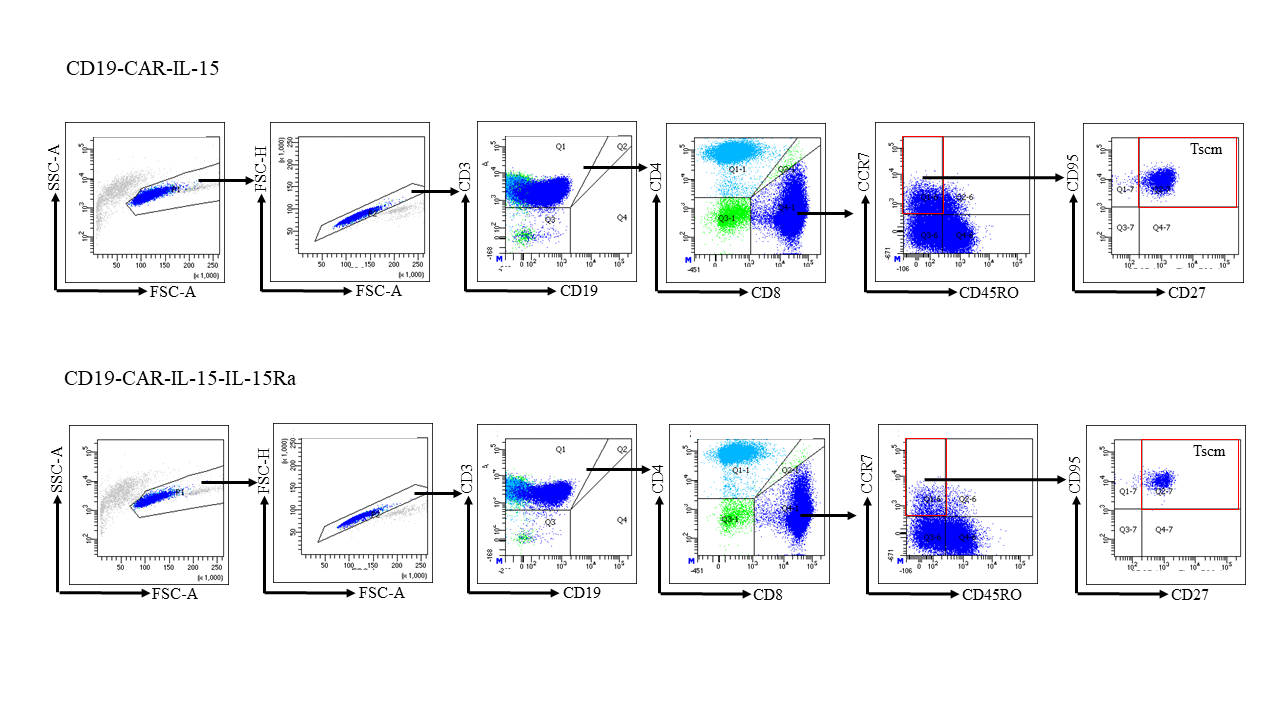


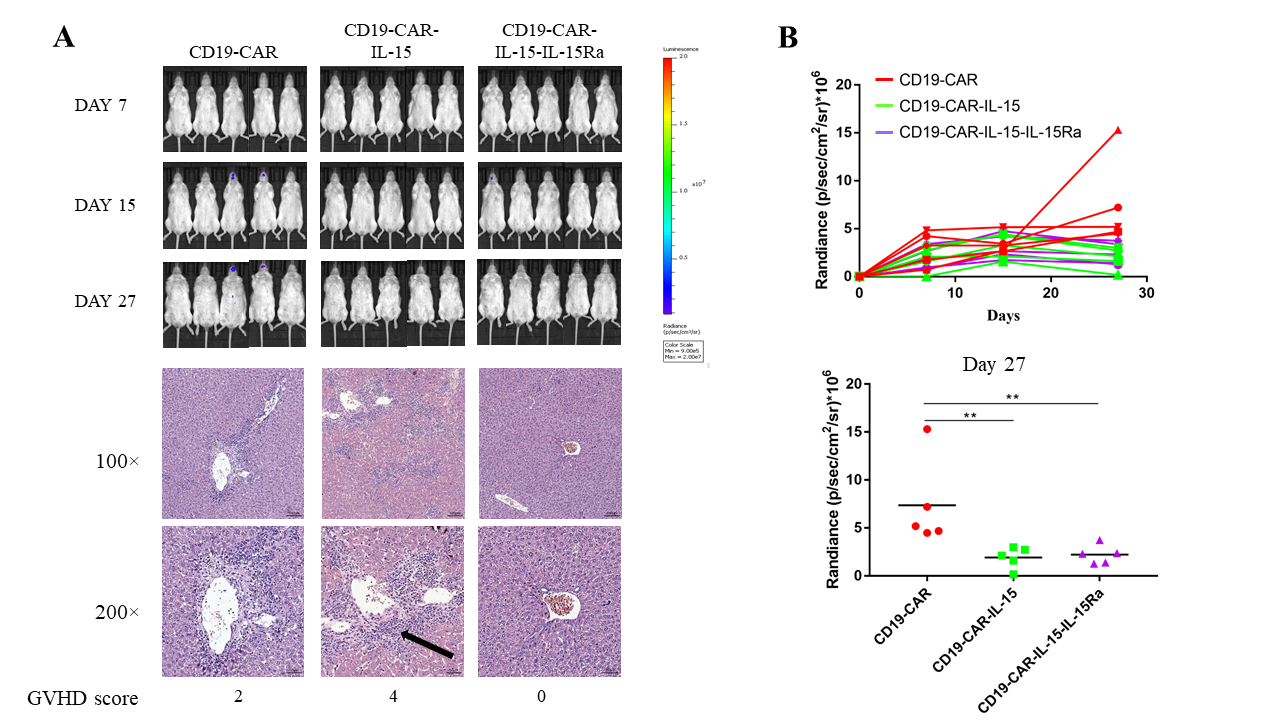
**Additional file Fig. 3**. 1 × 10^6^ NALM-6-eGFP cells were injected into NOD-SCID mice intravenously to construct the xenograft mouse model. One days after tumor cell injection, 1 × 10^7^ CAR-T cells (2 × 10^6^ CAR positive cells) were injected into tail vein once a day for three days. Tumor development was monitored using IVIS. (**A**) Upper penal shows quantitative bioluminescence (radiance=photons/cm^2^/sr) imaging data for all mice. Lower penal shows that livers from CAR-T treated mice (Day 27) were collected to stain hematoxylin and eosin. Black arrow shows the large area of necrotic lesions. (**B**) Statistic analysis of quantitative bioluminescence is shown. Results were analyzed by student’s *t*-test followed by Mann-Whitney test. ** *p* < 0.01.
